# Current perspectives on alphavirus encapsidation, assembly and budding

**DOI:** 10.3389/fcimb.2025.1719868

**Published:** 2026-01-05

**Authors:** Kanchan Bhardwaj, C. T. Ranjith-Kumar, Prasenjit Guchhait, Sudhanshu Vrati

**Affiliations:** 1 Department of Biotechnology, School of Engineering & Technology, Manav Rachna International Institute of Research and Studies, Faridabad, India; 2 Regional Centre for Biotechnology, National Capital Region Biotech Science Cluster, Faridabad, India; 3 University School of Biotechnology, Guru Gobind Singh Indraprastha University, New Delhi, India

**Keywords:** alphavirus, assembly, encapsidation, host factors, cellular membranes, cytoskeletal components

## Abstract

Alphaviruses are an escalating global concern due to their considerable clinical impact and expanding geographic distribution. Transmitted primarily through the bites of infected mosquitoes, alphaviruses cause a wide spectrum of arthritogenic and encephalitic diseases in both humans and animals. Their ability to re-emerge with enhanced fitness through adaptive mutations further underscores their public health importance. Despite advances in antiviral discovery and vaccine development, no licensed therapies are available for most of them, and vector control can only be partially effective. These limitations underscore the need for a mechanistic understanding of the virus life cycle to guide rational intervention strategies. Deciphering the molecular mechanisms of alphavirus assembly and budding has been a central research frontier. This perspective brings together the research on viral RNA encapsidation, structural elucidation of virus architecture, and the essential functions of host factors, membranes, and cytoskeletal components. An integrated understanding of the roles of both viral and host factors, along with the spatial and temporal coordination of events throughout the viral life cycle, is crucial for addressing key mechanistic gaps and for guiding the development of next-generation antiviral and vaccine strategies.

## 1 Introduction

Alphaviruses, members of the family *Togaviridae*, can infect a wide range of vertebrate hosts including humans, birds, large mammals, horses, rodents and fish (Weaver and Frolov, 2005; Forrester et al., 2012). Virus transmission typically occurs through arthropod vectors, primarily mosquitoes (Tesh et al., 1999; Braack et al., 2018). However, some alphaviruses deviate from this pattern, for example, Salmonid alphavirus is transmitted horizontally and through water contact, while Eilat virus (EILV), Taï Forest alphavirus (TALV), Mwinilunga virus (MWAV) and Agua Salud alphavirus (ASALV) are restricted to arthropod hosts (Deperasińska et al., 2018; Hermanns et al., 2020). More than 30 species of alphaviruses have been identified and classified into distinct complexes based on their antigenic properties (Weaver and Frolov, 2005). Traditionally, they have been grouped into Old World and New World lineages according to their geographic origin (Ahola et al., 2021; Kuhn, 2021). Old World alphaviruses typically cause fever, rash and arthritis, while New World alphaviruses are primarily associated with encephalitis, with the notable exception of Mayaro virus (MAYV), which is arthritogenic (Baxter and Heise, 2020; Zaid et al., 2021). Prominent arthritogenic alphaviruses include Ross River virus (RRV), Mayaro virus (MAYV), and O’nyong’nyong virus (ONNV) whereas the major encephalitic viruses are Eastern Equine Encephalitis Virus (EEEV), Western Equine Encephalitis Virus (WEEV), and Venezuelan Equine Encephalitis Virus (VEEV). Sindbis virus (SINV) and Semliki Forest Virus (SFV) can exhibit both arthritogenic and encephalitic characteristics, particularly in animal models (Baxter and Heise, 2020). Similarly, chikungunya virus (CHIKV), though primarily arthritogenic, has also been reported to cause encephalitis in humans (Mehta et al., 2018). Although, mortality rates associated with the arthritogenic alphavirus infection are low, these viruses are a serious public health concern and economic burden in low-income countries because in some cases, arthralgia and myalgia can persist for months to years following the acute phase of infection. Encephalitic alphavirus infection, on the other hand, can cause severe neurological diseases, often with high mortality and long-term sequelae (Baxter and Heise, 2020). Moreover, the combination of their ability to acquire adaptive mutations and the climate change-driven expansion of *Aedes* mosquito habitats has facilitated wider geographic spread of certain alphaviruses (Weaver et al., 2012; Farooq et al., 2025). A notable example is the E1-A226V mutation in CHIKV, which altered its vector specificity and contributed to its emergence as a global pathogen (Schuffenecker et al., 2006; Silva and Dermody, 2017). A similar mutation is found in SFV (Ahn et al., 1999). Although, several vaccine candidates are currently under development, only a few have received regulatory approval. Notably, FDA has recently approved a live attenuated vaccine (IXCHIQ) and a recombinant virus-like particle (VIMKUNYA), targeting CHIKV (Read et al., 2021; Hamer et al., 2025). Although, access to them is limited and live-attenuated vaccines have safety concerns. Despite these advances, no approved antiviral therapies or vaccines exist for most alphaviruses. Hence, scientific interest in studying alphaviruses remains strong, not only to develop effective antiviral strategies but also to advance our understanding of their fundamental biological processes.

Combined approaches including traditional virological methods and advanced structural biology and imaging techniques have yielded critical insights into key aspects of virus life cycle, including viral entry, genome replication, assembly, and release, while revealing the intricate interplay between the virus and host cellular machinery (Jose et al., 2009; Voss et al., 2010; Basore et al., 2019; Song et al., 2019; Holmes et al., 2020). Although, alphaviruses differ in receptor usage, tropism and pathogenicity, they share a conserved infection cycle (Jose et al., 2009; Song et al., 2019; Holmes et al., 2020). The alphavirus genome is a monopartite, linear, positive-sense single-stranded RNA (ssRNA) of approximately 11–12 kb in length, capped at the 5′ end and polyadenylated at the 3′ end. Belonging to Group IV of the Baltimore classification, the genomic RNA functions directly as mRNA for the synthesis of non-structural proteins and replicates *via* a complementary negative-sense RNA intermediate. This full-length negative-sense RNA also serves as the template for producing a shorter subgenomic RNA (26S RNA), transcribed from an internal promoter located downstream of the non-structural protein-coding region. The subgenomic RNA (sgRNA), which likewise contains a 5′ cap and 3′ poly(A) tail, directs the translation of the structural proteins, capsid, E3, E2, 6K, transframe (TF), and E1 as a single polyprotein (Barraza Sánchez et al., 2025). The capsid protein (CP) autoproteolytically cleaves itself from the polyprotein, inactivating its protease domain, and then it assembles with genomic RNA to form nucleocapsid cores (NC). The remaining structural polyprotein is directed to the endoplasmic reticulum (ER) *via* a signal sequence. Host proteases, including signalase and furin, process the individual proteins. Post-translational modifications, such as glycosylation, palmitoylation, and disulfide bond formation, are applied to the glycoproteins and TF protein as they move along the secretory pathway. Ultimately, mature glycoproteins, through a direct interaction between E2 and CP lead to virion budding. Viruses pinch off from the membrane in a scission step culminating in the release of infectious particles from the host cell. The replication and life cycle of alphaviruses were recently reviewed in detail by Luo et al. (2025).

High-resolution insights into the architecture of multiple alphaviruses have been obtained by advanced imaging methods, including cryogenic electron microscopy and tomography along with x-ray crystallography (Chmielewski et al., 2022; Lata et al., 2023; Comas-Garcia, 2024; Geng et al., 2025). The structure of a virus particle plays an active and essential role in ensuring the successful completion of its life cycle. Viral particles must perform several critical functions, including protecting the genome, specifically recognizing and packaging it, binding to host cell receptors to gain entry, and undergoing uncoating to deliver the genome to the appropriate site within the host cells. Deciphering the molecular mechanisms that enable alphaviruses to assemble and bud into such functional particles has long been a central focus of research. The fundamental questions that have been driving this field include: How is the viral genomic RNA (gRNA) selectively packaged? What drives nucleocapsid core formation? Where within the host cell does nucleocapsid assembly occur? And is nucleocapsid formation an independent precursor event, or a process tightly coupled with envelope acquisition? In this perspective, we describe these aspects and highlight the critical gaps that remain unresolved.

## 2 Genome encapsidation

**Figure 1:**
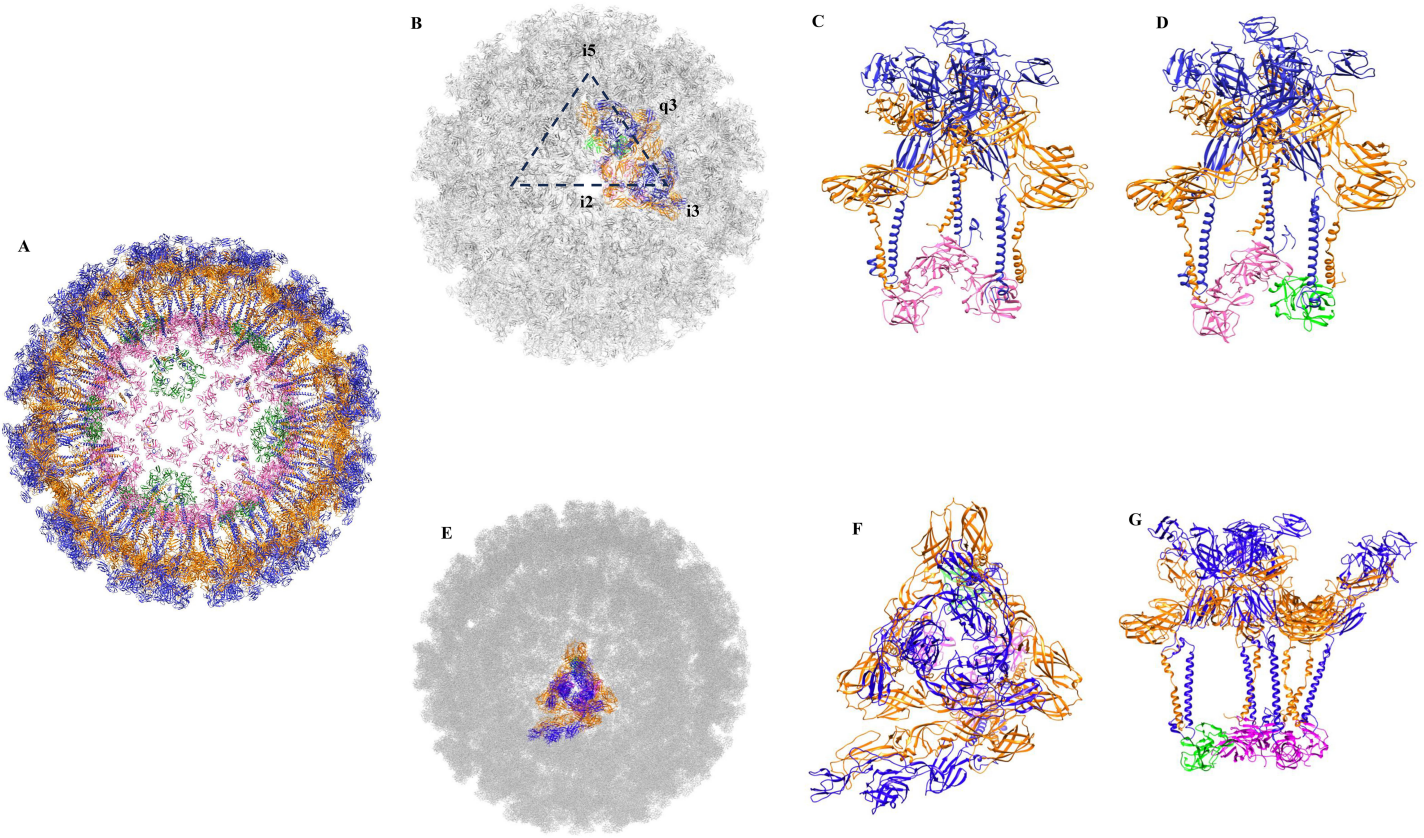
Architecture of chikungunya virus (pdb id: 6nk5). **(A)** Glycoproteins E1 and E2 are shown in orange and blue, respectively. Capsid protein pentamers and hexamers are shown in green and hot pink, respectively. **(B)** Arrangement of spikes on the virus surface, the 5-fold (i5) axis, 2-fold (i2) axis, icosahedral 3-fold (i3) and quasi-3-fold (q3) axis are shown. **(C)** Side-view of an i3 spike. **(D)** Side-view of a q3 spike. Capsid protein subunits involved in the formation of pentamers and hexamers are shown in green and hot pink, respectively. **(E)** An asymmetric unit of chikungunya virus. A single asymmetric unit on virus surface is shown in colour. **(F)** A top-view of an asymmetric unit. **(G)** A side-view of an asymmetric unit.

Alphavirus particles are assembled through coordinated interactions between their capsid protein, which packages the viral genomic RNA (gRNA) and the glycoproteins, E1 and E2, which are incorporated into the viral envelope. The spherical alphavirus particles are made of 20 triangular faces, 12 vertices and 30 edges with T = 4 icosahedral symmetries. A 3-fold vertex is located at the centre of each triangular face of the icosahedron. The virions have a diameter of 60–70 nm and are composed of three layers. Nucleocapsid core, the innermost layer has a diameter of around 40 nm and consists of 240 copies of the capsid protein bound to the viral RNA genome. Each capsid subunit uses its N-terminal domain to interact with the viral RNA genome. The 240 copies of the capsid protein are arranged with a quasi-equivalence of pentamer and hexamer units, which are assembled through slight rearrangements in subunit interactions (**Figure 1**). The middle layer is derived from the host cell plasma membrane. The outermost layer is composed of 80 spikes embedded within the host-derived lipid bilayer. Each spike is made up of trimers of heterodimers formed by the virus-encoded glycoproteins, E1 and E2 (**Figure 1**) (Kim et al., 2011; Kim et al., 2013; Geng et al., 2025) The outer glycoprotein layer is connected with the inner nucleocapsid core through interactions between the hydrophobic pocket in the C-terminal domain (CTD) of the capsid protein and the cytoplasmic tail of E2. The virions are formed of 60 asymmetric units (ASUs), and each ASU is formed by four E1/E2 heterodimers bound to four subunits of capsid protein (**Figure 1**). The spikes assemble on the icosahedral 3-fold (i3) and quasi-3-fold (q3) axes of symmetry. Thus, there are twenty i3 and sixty q3 spikes on a virus particle. Each of the 60 asymmetric units consist of one i3 E1/E2 heterodimer and a q3 trimer of E1/E2 heterodimers (**Figure 1**). The interactions among the glycoproteins E1 and E2 provide integrity and stability of the virus particles. Although glycoproteins experience different chemical environments within an asymmetric unit, both types of spikes are thought to be involved in virus entry, fusion and epitope accessibility.

To elucidate how the viral genomic RNA (gRNA) is selectively packaged and what drives nucleocapsid core formation, multiple studies have sought to identify distinct features within the viral RNA using diverse experimental approaches, including, *in vitro* binding assays, replicon systems and RNA packaging by helper viruses, sequence analysis of the RNA encapsidated by defective interfering (DI) particles that are formed at high multiplicity of infection and high-throughput techniques like CLIP-Seq (CrossLinking ImmunoPrecipitation-Sequencing) (Levis et al., 1986; Frolova et al., 1997; Volkova et al., 2006; Warrier et al., 2008; Kim et al., 2011; Kim et al., 2013; Atasheva et al., 2015; Sokoloski et al., 2017; Brown et al., 2020). Initial analyses using the replicon system and DI particles, showed that the nucleotide region between 945–1076 of SINV gRNA binds to capsid protein more efficiently than RNAs lacking these sequences, and that this region can enhance *in vitro* binding of non-viral RNA to the capsid (Levis et al., 1986; Frolova et al., 1997; Warrier et al., 2008). Similar packaging signals (PSs) were later identified in the nsP2 gene of SFV, chikungunya virus and RRV, consisting of four to six stem-loop structures with conserved GGG motifs at their bases. These PSs are located in the nsP1 gene of SINV and members of the VEEV complex (Volkova et al., 2006; Kim et al., 2011; Kim et al., 2013; Atasheva et al., 2015). While these studies provided compelling evidence for specific packaging signals within the alphavirus genome, they do not fully align with the observations that some DI RNAs lack these signals and that helper viruses do not always require them for packaging (Kim et al., 2011). In a subsequent analysis, Sokoloski et al. (2017) used CLIP-seq method and demonstrated that in cytoplasmic nucleocapsid complexes, SINV capsid protein binds most strongly within the last 1/3^rd^ of the viral gRNA (Sokoloski et al., 2017). However, mutations in these regions affected the stability of incoming viral RNA rather than nucleocapsid assembly or packaging (Sokoloski et al., 2017). Moreover, binding sites in mature virions were found to be non-specific (Sokoloski et al., 2017). In a complementary study with SFV, Brown et al. (2020) applied PAR-CLIP (photoactivatable ribonucleoside crosslinking and immunoprecipitation) method and identified 21 high-affinity CP-binding sites distributed across the first two-thirds of the genome, enriched in UUG and UGG-trinucleotide motifs and predicted to adopt stem-loop structures (Brown et al., 2020). Although, regions corresponding to the previously proposed PSs (2892–2926 and 2815-2856) were also detected, they exhibited weaker binding compared to many other regions. Mutations limited to these regions had little effect on virus production but simultaneous mutations of multiple sites impaired gRNA packaging and enhanced “nonspecific” sgRNA encapsidation. Comparison of cytoplasmic and virion-associated nucleocapsids revealed distinct CP-gRNA interaction patterns, suggesting conformational rearrangements during virus budding (Brown et al., 2020). This observation aligns with biochemical and structural evidence that nucleocapsid architecture dynamically remodels during maturation and release (Monroe and Schlesinger, 1983; Rümenapf et al., 1995; Rayaprolu et al., 2017).

Collectively, these studies have demonstrated that alphaviruses lack a distinct packaging signal, unlike certain other viruses such as the *psi* element in HIV, and that their nucleocapsid cores predominantly encapsidate full-length gRNA rather than the sgRNA or host cellular RNAs, despite the latter being present in high molar abundance within infected cells (Levis et al., 1986; Frolova et al., 1997; Volkova et al., 2006; Warrier et al., 2008; Kim et al., 2011; Kim et al., 2013; Atasheva et al., 2015; Sokoloski et al., 2017; Brown et al., 2020). An exception is the Aura virus, which packages both genomic and subgenomic RNAs to produce virions of approximately 72 nm and 62 nm in diameter (Rümenapf et al., 1995). Additional investigations have revealed that non-viral nucleic acids such as ssDNA and tRNA^asp^ can also drive nucleocapsid formation, although the length and composition of the encapsidated nucleic acid influence the stability and structural integrity of the resulting particles (Monroe and Schlesinger, 1983; Rayaprolu et al., 2017). Moreover, substitution of positively charged residues in the capsid with negatively charged ones result in the formation of empty core-like particles (Button and Mukhopadhyay, 2020). Interestingly, Kaelber et al. (2022) have reported that a minor subset of alphavirus particles can adopt a T = 3 icosahedral structure (Kaelber et al., 2022). Such structural plasticity, manifested as a variation in triangulation number and a degree of promiscuity in RNA encapsidation highlights the inherent adaptability of alphavirus assembly and its potential utility in the rational design of alphavirus-based vaccines.

## 3 Virus assembly and budding

Another key question at the forefront of alphavirus research has been whether nucleocapsid formation occurs as an independent precursor event or is highly coupled with envelope acquisition during virion assembly. Two major models have been proposed to explain the assembly of infectious alphavirus particles: (i) the nucleocapsid-directed assembly model and (ii) the glycoprotein-directed assembly model (Lazaro et al., 2018; Button and Mukhopadhyay, 2021). In the nucleocapsid-directed model, binding of viral RNA to the capsid protein triggers interactions among capsid subunits, leading to the formation of cytoplasmic nucleocapsid cores. These preassembled cores are thought to act as scaffolds for wrapping them up with an envelope and define virion geometry (Acheson and Tamm, 1967; Suomalainen et al., 1992; Owen and Kuhn, 1997; Hernandez et al., 2000; Soonsawad et al., 2010; Jose et al., 2012; Snyder et al., 2012; Button and Mukhopadhyay, 2021). In contrast, the glycoprotein-directed model proposes that RNA-capsid protein interactions promote engagement of the cytoplasmic tail of the E2 glycoprotein with the hydrophobic pocket of the capsid protein C-terminal domain, enabling virion assembly directly at the plasma membrane without requiring preformed cores (Forsell et al., 1995; Forsell et al., 1996; Lee et al., 1996; Skoging et al., 1996; Skoging-Nyberg and Liljestrom, 2001; Lulla et al., 2013). Evidence support both models, suggesting that alphaviruses may employ flexible or context-dependent strategies to ensure efficient RNA encapsidation and virion formation. Notably, Ruiz-Guillen et al. (2016) have reported the budding of capsid-deficient infectious alphavirus microvesicles from cells lacking capsid gene expression, indicating that glycoprotein spikes alone can drive virus budding, independently of nucleocapsid formation or capsid and E2 interactions (Ruiz-Guillen et al., 2016). Supporting this, cryo-ET studies on chikungunya virus have shown that lateral interactions among glycoprotein spikes are essential for budding, and disruption of these interactions inhibits the process (Chmielewski et al., 2022). These analyses have further revealed that during budding non-icosahedral nucleocapsids can act as scaffolds that promote the formation of icosahedral spike lattice, which subsequently reshapes the nucleocapsids into ordered icosahedral cores (Chmielewski et al., 2022). Collectively, these findings suggest that alphavirus budding can proceed without nucleocapsid formation and virion geometry is not exclusively determined by the NC. However, the absence of NC at the budding site result in formation of polydisperse particles, likely due to altered assembly kinetics and loss of coordination with the scission machinery (Lazaro et al., 2018). Although evidence for the alphavirus scission process remain scanty and require further studies. Scission of the bud formed as a result of viral glycoprotein lattice-driven membrane curvature and neck constriction could be mediated either by host ESCRT machinery, as in HIV or by viral factors, analogous to the M2 protein in influenza virus (Taylor et al., 2007; Torii et al., 2020). SFV is shown to bud independently of VPS4, indicating an ESCRT-independent mechanism, whereas ESCRT factors are recruited during chikungunya virus replication (Taylor et al., 2007; Torii et al., 2020).

Beyond the mechanistic aspects, an important area of investigation has been to understand what determines the molecular composition of alphavirus particles and how variations in virions produced by vertebrate versus arthropod hosts influence their infectivity, stability and host adaptation. In addition to the capsid and envelope glycoproteins E1 and E2, other structural proteins encoded by the viral sgRNA and host factors have also been shown to play important roles in assembly and maturation. E3, for instance, assists in E1-E2 heterodimerization and stabilizes the spike complex by clamping the two glycoproteins together during their transport through the secretory pathway, thereby preventing premature exposure of the fusion loop in acidic environments (Snyder and Mukhopadhyay, 2012; Uchime et al., 2013; Fields and Kielian, 2015). E2 and E3 are synthesized as a precursor protein named, p62. Prior to virion release, furin-mediated cleavage of the precursor protein, p62 in the late Golgi separates E2 from E3, yielding mature E1-E2 heterodimers. Although SFV and VEEV retain E3 transiently in mature virions, its subsequent release is required for membrane fusion and host cell entry (Zhang et al., 2003; Zhang and Kielian, 2004; Wu et al., 2007; Zhang et al., 2011). The TransFrame (TF) protein, produced *via* a programmed ribosomal frameshift during translation of the 6K gene, shares an N-terminal sequence with 6K but possesses a unique C-terminal region. While 6K functions as a viroporin facilitating virion release by forming ion channels, TF undergoes palmitoylation and contributes to virion maturation (Ramsey and Mukhopadhyay, 2017; Ramsey et al., 2017; Dey et al., 2019). Our understanding of the roles of 6K and TF proteins is still developing, as recently reviewed by Negi et al. (2025). The review underscores current gaps, particularly the absence of high-resolution structural data for 6K/TF, incomplete understanding of their ion-channel functions in infected cells and their interplay with host factors and emphasizes that comparative analyses across viroporins can inform the design of common inhibitors that may have utility against alphaviruses and other viruses.

Alphaviruses can also incorporate host-derived molecules into their particles, reflecting the influence of the host cell environment. Proteomic analyses have detected ribosomal 40S small subunit components in arthropod-derived particles and in a small fraction of vertebrate-derived particles (Sokoloski et al., 2013). Interestingly, such particles with the ribosomal subunit components exhibit higher specific infectivity and trigger a weak type I interferon response (Sokoloski et al., 2013). Beyond this, alphaviruses produced in arthropod versus vertebrate cells show notable differences in genomic RNA modifications and in the lipid and glycan composition of their envelopes, while no major differences are noted in the amount of viral proteins found in the virus particles (Kalvodova et al., 2009; Knight et al., 2009; Dunbar et al., 2019; Crawford et al., 2022). Virions derived from vertebrate cells incorporate more long-chain fatty acid-containing lipids, making them denser than their arthropod-derived counterparts (Kalvodova et al., 2009; Dunbar et al., 2019). Host-specific glycosylation patterns on envelope glycoproteins significantly influence infection efficiency and host adaptation (Knight et al., 2009). Although limited, recent studies have begun to elucidate the host-dependent differences in virion composition and function (Kalvodova et al., 2009; Knight et al., 2009; Crawford et al., 2022; Chen et al., 2024; Ventoso et al., 2024; Wang et al., 2024).

## 4 Spatial and temporal coordination of nucleocapsid formation, virus assembly and release

Similar to other positive-sense RNA viruses, alphavirus replication and assembly are associated with rearrangement and restructuring of cellular membranes and the cytoskeleton that support viral processes. Infected cells exhibit distinct ultrastructural features including replication spherules, cytopathic vacuoles (CPV-I and CPV-II) and both long and short filopodial extensions (Elmasri et al., 2021). The 50–60 nm replication spherules, found on the plasma membrane and the membranes of CPV-I, serve as the primary sites for viral genome replication (Frolova et al., 2010). Cytopathic vacuoles, CPV-I are single-membrane structures (0.6-2 μm diameter) that originate from endosomal and lysosomal limiting membranes while CPV-II, appearing about four hours post-infection, are single- or double-membrane vesicles (100–400 nm by 1-2 μm), derived from the trans-Golgi network (TGN) (Froshauer et al., 1988; Zhao et al., 1994; Soonsawad et al., 2010; Brown et al., 2018). ADP-ribosylation factor 1 (Arf1) is a host factor indicated to play a role in the biogenesis of CPV-II (Brown et al., 2018). Single-membrane CPV-II typically have nucleocapsids (NCs) attached to their cytoplasmic face, whereas double-membrane CPV-II contain NCs on both sides and also harbour viral glycoproteins E1 and E2 (Griffiths et al., 1983; Jose et al., 2017). The vacuoles are trafficked through the cytoskeletal actin, involving host factors, RAS-related C3 botulinum toxin substrate 1 (Rac1), actin-related protein-3 (Arp3), and Phosphatidylinositol-4-Phosphate 5-Kinase Type 1 Alpha (PIP5K1-α) (Brown et al., 2018). In mosquito host cells, where alphaviruses establish persistent, noncytopathic infection, large cytopathic vacuoles with features intermediate between CPV-I and CPV-II are observed throughout infection. These structures contain replication spherules similar to CPV-I, as well as viral glycoproteins on the inside and nucleocapsids on the outside, similar to CPV-II. Internally budded virions within these vacuoles are transported to adjacent uninfected cells via long filopodial extensions (Jose et al., 2017). Notably, mosquito cells produce virus particles at a steady rate but with significantly reduced growth rates as compared to the vertebrate cells.

Alphavirus budding occurs at the plasma membrane, including but not exclusively through specialized domains such as short filopodial extensions (2-7 μm long), which are composed mainly of actin filaments. The precise role of these extensions in assembly and budding remains unclear, although they may also contribute to pathogenesis (Ahola et al., 2000). In contrast, long filopodial extensions (10-60 μm) are composed of both actin and microtubules. They are observed in multiple cell types, including epithelial cells, fibroblasts and mosquito cells infected with alphaviruses such as SINV, SFV, CHIKV and VEEV (Martinez et al., 2014; Martinez and Kielian, 2016; Jose et al., 2017). Viral proteins, E1, E2 and capsid protein are distributed along these long extensions, suggesting a role in promoting efficient cell-to-cell spread by positioning newly released virions in close proximity of uninfected cells, thereby evading immune detection without inducing cellular merging (Lee et al., 2011).

**Figure 2:**
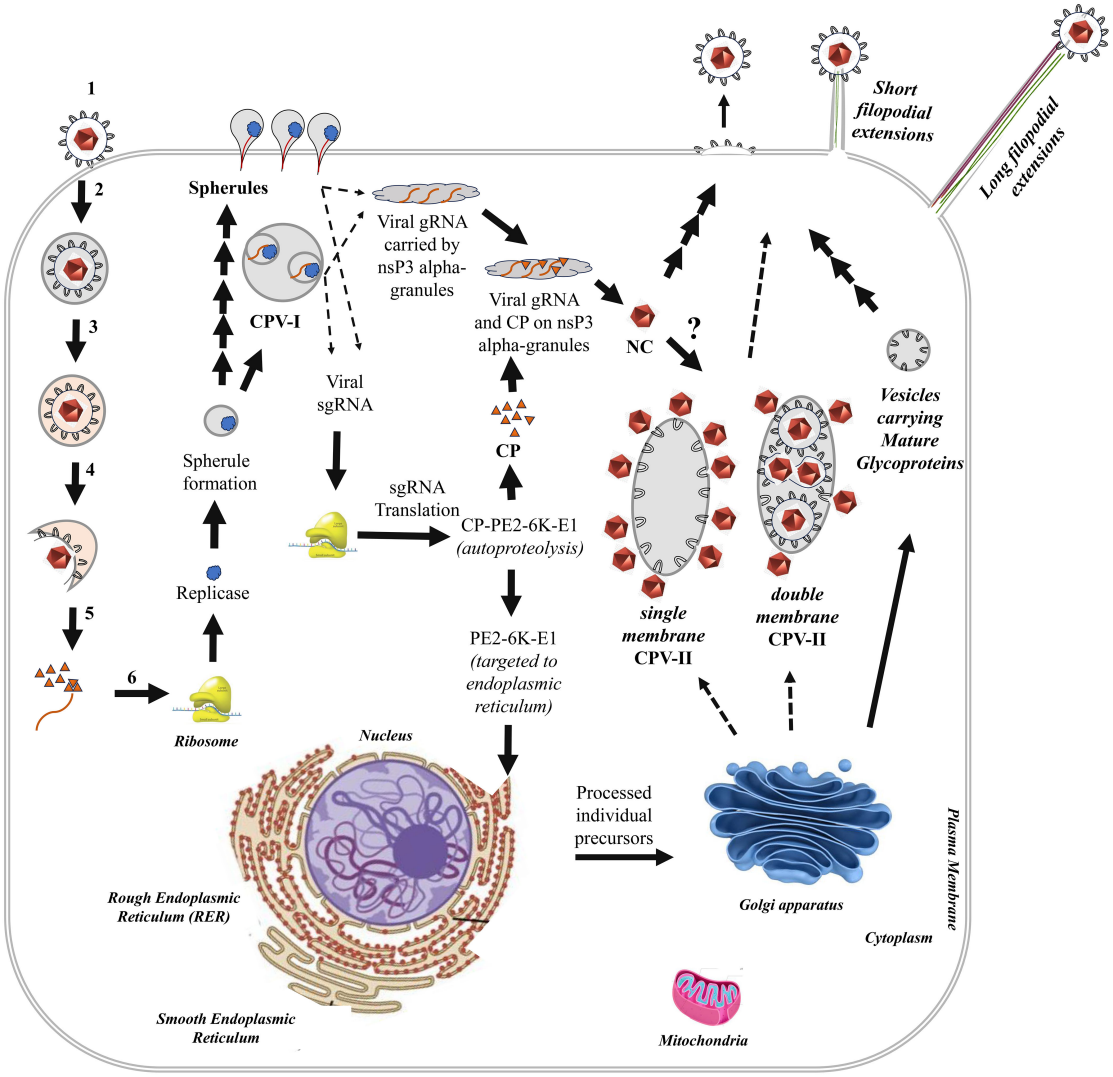
Schematic representation of the alphavirus life cycle in a vertebrate host cell. The left side of the cell illustrates the initial six steps of the virus life cycle: 1. Virus Attachment; 2. Virus Internalization; 3. Endosome Acidification; 4. Membrane Fusion; 5. Nucleocapsid Core (NC) Release & Disassembly; 6. Replicase Synthesis. Arrows indicate the sequential progression and connectivity of subsequent steps in the life cycle. Major cellular organelles, viral components and virus-induced structures characteristic of alphavirus-infected cells are labelled.

How and where the viral genomic RNA and capsid protein converge to initiate nucleocapsid formation has been a central question. In a recent study Kril et al. (2024) demonstrated co-localization of viral gRNA and capsid protein on nsP3 alpha-granules, proposing that nsP3 alpha-granules may transport viral RNA from replication spherules and deliver it to capsid proteins in the cytoplasm to facilitate nucleocapsid formation (Kril et al., 2024). Further studies are needed to elucidate the mechanisms by which preassembled nucleocapsids are recruited to the plasma membrane or acquired by CPV-II during subsequent stages of virion formation. Based on the precedence from other viruses, the host factor Arf1, known to mediate biogenesis of CPV-II, may play an important role. Both, SARS-CoV and HCV manipulate Arf1 to induce fragmentation of the Golgi (Hansen et al., 2017; Gonzales and Machamer, 2021). Co-localization of Golgi vesicles with HCV replication site has also been observed (Hansen et al., 2017). Similar mechanisms can be speculated to operate in alphaviruses but remains to be understood completely (Sengupta et al., 2022). A schematic representation of the alphavirus life cycle in a vertebrate host cell is depicted in **Figure 2**.

## 5 Concluding remarks

Alphaviruses exhibit remarkable selectivity in packaging their full-length genomic RNA, yet there is also evidence suggesting an unexpected flexibility in their assembly process. The ability of non-viral nucleic acids, such as ssDNA and tRNA^asp^, to drive nucleocapsid formation and the potential for alternative triangulation numbers indicate that alphavirus assembly may tolerate a level of promiscuity. Such mechanistic insights into alphavirus particle biology are guiding the design of safe and more immunogenic vaccines, ranging from VLPs to self-amplifying RNA/alphavirus vector platforms (Hikke et al., 2016; Francica et al., 2021; Zhang et al., 2025). Despite significant progress, several key questions remain unanswered. For instance, why do virion populations exhibit such heterogeneity, manifested as defective interfering (DI) particles and variations in lipid, glycan, and protein composition, and is this variability adaptive or merely stochastic? The implications of this heterogeneity for infectivity, immune evasion, and transmission dynamics are largely unexplored but may hold critical insights for pathogenesis and vaccine design. In addition, the roles of host factors and intracellular trafficking pathways in modulating particle assembly, release and infectivity remain incompletely defined, particularly in the context of the distinct replication environments of vertebrate and arthropod hosts. Understanding these host-specific interactions will be essential to elucidate the mechanisms driving alphavirus persistence and transmission. Finally, the impact of virion composition, lipid milieu, and structural variability on host range and tissue tropism remains an underexplored but critical frontier. Future studies should aim to dissect these determinants, thereby uncovering novel antiviral targets and informing the rational design of next-generation alphavirus vaccines and virus-like particle (VLP) platforms.

## Data Availability

The original contributions presented in the study are included in the article/supplementary material. Further inquiries can be directed to the corresponding author.

## References

[B49] Acheson N. H. Tamm I. (1967). Replication of Semliki Forest virus: an electron microscopic study. Virology. 32, 128–143. doi: 10.1016/0042-6822(67)90261-9, PMID: 6067298

[B16] Ahn A. Schoepp R. J. Sternberg D. Kielian M. (1999). Growth and stability of a cholesterol-independent Semliki Forest virus mutant in mosquitoes. Virology. 262, 452–456. doi: 10.1006/viro.1999.9932, PMID: 10502523

[B90] Ahola T. Kujala P. Tuittila M. Blom T. Laakkonen P. Hinkkanen A. . (2000). Effects of palmitoylation of replicase protein nsP1 on alphavirus infection. J. Virol. 74, 6725–6733. doi: 10.1128/JVI.74.15.6725-6733.2000, PMID: 10888610 PMC112188

[B8] Ahola T. McInerney G. Merits A. (2021). “ Alphavirus RNA replication in vertebrate cells,” in Adv. Virus Res. 111, 111–156. doi: 10.1016/bs.aivir.2021.07.003, PMID: 34663497

[B36] Atasheva S. Kim D. Y. Frolova E. I. Frolov I. (2015). Venezuelan equine encephalitis virus variants lacking transcription inhibitory functions demonstrate highly attenuated phenotype. J. virology. 89, 71–82. doi: 10.1128/JVI.02252-14, PMID: 25320296 PMC4301144

[B24] Barraza Sánchez J. J. Volpe S. Faraj S. E. Filomatori C. V. (2025). Functional RNA elements in alphavirus genomes. Curr. Microbiol. 82, 364. doi: 10.1007/s00284-025-04364-1, PMID: 40616665

[B22] Basore K. Kim A. S. Nelson C. A. Zhang R. Smith B. K. Uranga C. . (2019). Cryo-EM structure of chikungunya virus in complex with the mxra8 receptor. Cell. 177, 1725–1737.e16. doi: 10.1016/j.cell.2019.04.006, PMID: 31080061 PMC7227486

[B9] Baxter V. K. Heise M. T. (2020). Immunopathogenesis of alphaviruses. Adv. Virus Res. 107, 315–382. doi: 10.1016/bs.aivir.2020.06.002, PMID: 32711733 PMC8224468

[B3] Braack L. Gouveia de Almeida A. P. Cornel A. J. Swanepoel R. de Jager C. (2018). Mosquito-borne arboviruses of African origin: review of key viruses and vectors. Parasit Vectors. 11, 29. doi: 10.1186/s13071-017-2559-9, PMID: 29316963 PMC5759361

[B38] Brown R. S. Anastasakis D. G. Hafner M. Kielian M. (2020). Multiple capsid protein binding sites mediate selective packaging of the alphavirus genomic RNA. Nat. Commun. 11, 4693. doi: 10.1038/s41467-020-18447-z, PMID: 32943634 PMC7499256

[B86] Brown R. S. Wan J. J. Kielian M. (2018). The alphavirus exit pathway: what we know and what we wish we knew. Viruses. 10, 89. doi: 10.3390/v10020089, PMID: 29470397 PMC5850396

[B42] Button J. M. Mukhopadhyay S. (2020). Removing the polyanionic cargo requirement for assembly of alphavirus core-like particles to make an empty alphavirus core. Viruses 12, 846. doi: 10.3390/v12080846, PMID: 32756493 PMC7472333

[B45] Button J. M. Mukhopadhyay S. (2021). Capsid-E2 interactions rescue core assembly in viruses that cannot form cytoplasmic nucleocapsid cores. J. Virol. 95, e0106221. doi: 10.1128/JVI.01062-21, PMID: 34495691 PMC8549512

[B79] Chen R. Wang Z. Zhang L. (2024). Research trends on alphavirus receptors: a bibliometric analysis. Front. Cell Infect. Microbiol. 14. doi: 10.3389/fcimb.2024.1388360, PMID: 38841111 PMC11150648

[B27] Chmielewski D. Schmid M. F. Simmons G. Jin J. Chiu W. (2022). Chikungunya virus assembly and budding visualized in *situ* using cryogenic electron tomography. Nat. Microbiol. 7, 1270–1279. doi: 10.1038/s41564-022-01164-2, PMID: 35773421 PMC9930444

[B29] Comas-Garcia M. (2024). How structural biology has changed our understanding of icosahedral viruses. J. Virol. 98, e01111–e01123. doi: 10.1128/jvi.01111-23, PMID: 39291975 PMC11495149

[B75] Crawford J. M. Yan L. L. Zaher H. Hardy R. W. (2022). Host-dependent modifications of packaged alphavirus genomic RNA influence virus replication in mammalian cells. Viruses. 14, 2606. doi: 10.3390/v14122606, PMID: 36560610 PMC9781491

[B5] Deperasińska I. Schulz P. Siwicki A. K. (2018). Salmonid alphavirus (SAV). J. Vet. Res. 62, 1–6. doi: 10.2478/jvetres-2018-0001, PMID: 29978121 PMC5957455

[B70] Dey D. Siddiqui S. I. Mamidi P. Ghosh S. Kumar C. S. Chattopadhyay S. . (2019). The effect of amantadine on an ion channel protein from Chikungunya virus. PloS Negl. Trop. Dis. 13, e0007548. doi: 10.1371/journal.pntd.0007548, PMID: 31339886 PMC6655611

[B74] Dunbar C. A. Rayaprolu V. Wang J. C. Brown C. J. Leishman E. Jones-Burrage S. . (2019). Dissecting the components of sindbis virus from arthropod and vertebrate hosts: implications for infectivity differences. ACS Infect. Dis. 5, 892–902. doi: 10.1021/acsinfecdis.8b00356, PMID: 30986033 PMC6570550

[B81] Elmasri Z. Nasal B. L. Jose J. (2021). Alphavirus-induced membrane rearrangements during replication, assembly, and budding. Pathogens. 10, 984. doi: 10.3390/pathogens10080984, PMID: 34451448 PMC8399458

[B13] Farooq Z. Segelmark L. Rocklöv J. Lillepold K. Sewe M. O. Briet O. J. T. . (2025). Impact of climate and *Aedes albopictus* establishment on dengue and chikungunya outbreaks in Europe: a time-to-event analysis. Lancet Planetary Health 9, e374–e383. doi: 10.1016/S2542-5196(25)00059-2, PMID: 40381632

[B64] Fields W. Kielian M. (2015). Interactions involved in pH protection of the alphavirus fusion protein. Virology. 486, 173–179. doi: 10.1016/j.virol.2015.08.028, PMID: 26433749 PMC4679575

[B2] Forrester N. L. Palacios G. Tesh R. B. Savji N. Guzman H. Sherman M. . (2012). Genome-scale phylogeny of the alphavirus genus suggests a marine origin. J. Virol. 86, 2729–2738. doi: 10.1128/JVI.05591-11, PMID: 22190718 PMC3302268

[B57] Forsell K. Griffiths G. Garoff H. (1996). Preformed cytoplasmic nucleocapsids are not necessary for alphavirus budding. EMBO J. 15, 6495–6505. doi: 10.1002/j.1460-2075.1996.tb01040.x, PMID: 8978676 PMC452474

[B56] Forsell K. Suomalainen M. Garoff H. (1995). Structure-function relation of the NH2-terminal domain of the Semliki Forest virus capsid protein. J. Virol. 69, 1556–1563. doi: 10.1128/jvi.69.3.1556-1563.1995, PMID: 7853489 PMC188749

[B99] Francica J. R. Shi W. Chuang G.-Y. Chen S. J. Da Silva Pereira L. Farney S. K. . (2021). Design of alphavirus virus-like particles presenting circumsporozoite junctional epitopes that elicit protection against malaria. Vaccines. 9, 272. doi: 10.3390/vaccines9030272, PMID: 33803622 PMC8003078

[B30] Frolova E. Frolov I. Schlesinger S. (1997). Packaging signals in alphaviruses. J. virology. 71, 248–258. doi: 10.1128/jvi.71.1.248-258.1997, PMID: 8985344 PMC191045

[B82] Frolova E. I. Gorchakov R. Pereboeva L. Atasheva S. Frolov I. (2010). Functional Sindbis virus replicative complexes are formed at the plasma membrane. J. Virol. 84, 11679–11695. doi: 10.1128/JVI.01441-10, PMID: 20826696 PMC2977861

[B83] Froshauer S. Kartenbeck J. Helenius A. (1988). Alphavirus RNA replicase is located on the cytoplasmic surface of endosomes and lysosomes. J. Cell Biol. 107, 2075–2086. doi: 10.1083/jcb.107.6.2075, PMID: 2904446 PMC2115628

[B28] Geng Q. Navaratnarajah C. K. Zhang W. (2025). Structural and functional hallmarks of sindbis virus proteins: from virion architecture to pathogenesis. Int. J. Mol. Sci. 26, 8323. doi: 10.3390/ijms26178323, PMID: 40943245 PMC12428335

[B95] Gonzales R. R. Machamer C. E. (2021). The SARS CoV-1 3a protein disrupts Golgi complex morphology and cargo trafficking. bioRxiv. doi: 10.1101/2021.04.19.440492

[B88] Griffiths G. Simons K. Warren G. Tokuyasu K. T. (1983). Immunoelectron microscopy using thin, frozen sections: application to studies of the intracellular transport of Semliki Forest virus spike glycoproteins. Methods Enzymol. 96, 466–485. doi: 10.1016/S0076-6879(83)96041-X, PMID: 6656640

[B18] Hamer M. J. McCarty J. M. Pierson B. C. Regules J. A. Mendy J. Sanborn A. D. . (2025). Safety and immunogenicity of an adjuvanted chikungunya virus virus-like particle (CHIKV VLP) vaccine in previous recipients of other alphavirus vaccines versus alphavirus vaccine-naive controls: an open-label, parallel-group, age-matched, sex-matched, phase 2 randomised controlled study. Lancet Microbe 6, 101000. doi: 10.1016/j.lanmic.2024.101000, PMID: 39954701

[B96] Hansen M. D. Johnsen I. B. Stiberg K. A. Sherstova T. Wakita T. Richard G. M. . (2017). Hepatitis C virus triggers golgi fragmentation and autophagy through the immunity-related GTPase M. Proc. Natl. Acad. Sci. U.S.A. 114, E3462–E3471. doi: 10.1073/pnas.1616683114, PMID: 28389568 PMC5410803

[B6] Hermanns K. Marklewitz M. Zirkel F. Overheul G. J. Page R. A. Loaiza J. R. . (2020). Agua Salud alphavirus defines a novel lineage of insect-specific alphaviruses discovered in the New World. J. Gen. Virol. 101, 96–104. doi: 10.1099/jgv.0.001344, PMID: 31674898 PMC7414432

[B51] Hernandez R. Lee H. Nelson C. Brown D. T. (2000). A single deletion in the membrane-proximal region of the Sindbis virus glycoprotein E2 endodomain blocks virus assembly. J. Virol. 74, 4220–4228. doi: 10.1128/JVI.74.9.4220-4228.2000, PMID: 10756035 PMC111937

[B98] Hikke M. C. Geertsema C. Wu V. Metz S. W. van Lent J. W. Vlak J. M. . (2016). Alphavirus capsid proteins self-assemble into core-like particles in insect cells: A promising platform for nanoparticle vaccine development. Biotechnol. J. 11, 266–273. doi: 10.1002/biot.201500147, PMID: 26287127

[B20] Holmes A. C. Basore K. Fremont D. H. Diamond M. S. A. (2020). Molecular understanding of alphavirus entry. PloS Pathog. 16, e1008876. doi: 10.1371/journal.ppat.1008876, PMID: 33091085 PMC7580943

[B46] Jose J. Przybyla L. Edwards T. J. Perera R. Burgner J. W. 2nd Kuhn R. J. (2012). Interactions of the cytoplasmic domain of Sindbis virus E2 with nucleocapsid cores promote alphavirus budding. J. Virol. 86, 2585–2599. doi: 10.1128/JVI.05860-11, PMID: 22190727 PMC3302261

[B19] Jose J. Snyder J. E. Kuhn R. J. A. (2009). Structural and functional perspective of alphavirus replication and assembly. Future Microbiol. 4, 837–856. doi: 10.2217/fmb.09.59, PMID: 19722838 PMC2762864

[B87] Jose J. Taylor A. B. Kuhn R. J. (2017). Spatial and temporal analysis of alphavirus replication and assembly in mammalian and mosquito cells. mBio. 8, e02294–e02216. doi: 10.1128/mBio.02294-16, PMID: 28196962 PMC5312085

[B43] Kaelber J. T. Chmielewski D. Chiu W. Auguste A. J. (2022). Alphavirus particles can assemble with an alternate triangulation number. Viruses. 14, 2650. doi: 10.3390/v14122650, PMID: 36560655 PMC9780915

[B76] Kalvodova L. Sampaio J. L. Cordo S. Ejsing C. S. Shevchenko A. Simons K. (2009). The lipidomes of vesicular stomatitis virus, semliki forest virus, and the host plasma membrane analyzed by quantitative shotgun mass spectrometry. J. Virol. 83, 7996–8003. doi: 10.1128/JVI.00635-09, PMID: 19474104 PMC2715782

[B35] Kim D. Y. Atasheva S. Frolova E. I. Frolov I. (2013). Venezuelan equine encephalitis virus nsP2 protein regulates packaging of the viral genome into infectious virions. J. virology. 87, 4202–4213. doi: 10.1128/JVI.03142-12, PMID: 23365438 PMC3624340

[B34] Kim D. Y. Firth A. E. Atasheva S. Frolova E. I. Frolov I. (2011). Conservation of a packaging signal and the viral genome RNA packaging mechanism in alphavirus evolution. J. virology. 85, 8022–8036. doi: 10.1128/JVI.00644-11, PMID: 21680508 PMC3147971

[B77] Knight R. L. Schultz K. L. Kent R. J. Venkatesan M. Griffin D. E. (2009). Role of N-linked glycosylation for sindbis virus infection and replication in vertebrate and invertebrate systems. J. Virol. 83, 5640–5647. doi: 10.1128/JVI.02427-08, PMID: 19297464 PMC2681937

[B94] Kril V. Hons M. Amadori C. Zimberger C. Couture L. Bouery Y. . (2024). Alphavirus nsP3 organizes into tubular scaffolds essential for infection and the cytoplasmic granule architecture. Nat. Commun. 15, 8106. doi: 10.1038/s41467-024-51952-z, PMID: 39285216 PMC11405681

[B7] Kuhn R. J. (2021). “ Togaviridae: the viruses and their replication,” in Fields Virology: Emerging Viruses, 7th ed, vol. 1 . Eds. Howley P. M. Knipe D. M. ( Lippincott Williams & Wilkins, Philadelphia, PA, USA), 170–193.

[B26] Lata K. Charles S. Prasad V. M. (2023). Advances in computational approaches to structure determination of alphaviruses and flaviviruses using cryo-electron microscopy. J. Struct. Biol. 215, 107993. doi: 10.1016/j.jsb.2023.107993, PMID: 37414374

[B44] Lazaro G. R. Mukhopadhyay S. Hagan M. F. (2018). Why enveloped viruses need cores—the contribution of a nucleocapsid core to viral budding. Biophys. J. 114, 619–630. doi: 10.1016/j.bpj.2017.11.3782, PMID: 29414708 PMC5985022

[B93] Lee C. Y. Kam Y. W. Fric J. Malleret B. Koh E. G. Prakash C. . (2011). Chikungunya virus neutralization antigens and direct cell-to-cell transmission are revealed by human antibody-escape mutants. PloS Pathog. 7, e1002390. doi: 10.1371/journal.ppat.1002390, PMID: 22144891 PMC3228792

[B53] Lee S. Owen K. E. Choi H. K. Lee H. Lu G. Wengler G. . (1996). Identification of a protein binding site on the surface of the alphavirus nucleocapsid and its implication in virus assembly. Structure 4, 531–541. doi: 10.1016/S0969-2126(96)00059-7, PMID: 8736552

[B31] Levis R. Weiss B. G. Tsiang M. Huang H. Schlesinger S. (1986). Deletion mapping of Sindbis virus DI RNAs derived from cDNAs defines the sequences essential for replication and packaging. Cell. 44, 137–145. doi: 10.1016/0092-8674(86)90492-7, PMID: 3753584

[B55] Lulla V. Kim D. Y. Frolova E. I. Frolov I. (2013). The amino-terminal domain of alphavirus capsid protein is dispensable for viral particle assembly but regulates RNA encapsidation through cooperative functions of its subdomains. J. Virol. 87, 12003–12019. doi: 10.1128/JVI.01960-13, PMID: 24006447 PMC3807903

[B25] Luo D. Tan Y. B. Law M. C. Y. Jin J. (2025). A structural perspective on the alphavirus life cycle. Annu. Rev. Virol. 12, 299–314. doi: 10.1146/annurev-virology-093022-010359, PMID: 40720808

[B91] Martinez M. G. Kielian M. (2016). Intercellular extensions are induced by the alphavirus structural proteins and mediate virus transmission. PloS Pathog. 12, e1006061. doi: 10.1371/journal.ppat.1006061, PMID: 27977778 PMC5158078

[B92] Martinez M. G. Snapp E. L. Perumal G. S. Macaluso F. P. Kielian M. (2014). Imaging the alphavirus exit pathway. J. Virol. 88, 6922–6933. doi: 10.1128/JVI.00592-14, PMID: 24696489 PMC4054368

[B11] Mehta R. Gerardin P. de Brito C. A. A. Soares C. N. Ferreira M. L. B. Solomon T. (2018). The neurological complications of chikungunya virus: A systematic review. Rev. Med. Virol. 28, e1978. doi: 10.1002/rmv.1978, PMID: 29671914 PMC5969245

[B40] Monroe S. S. Schlesinger S. (1983). RNAs from two independently isolated defective interfering particles of Sindbis virus contain a cellular tRNA sequence at their 5’ ends. Proc. Natl. Acad. Sci. U S A. 80, 3279–3283. doi: 10.1073/pnas.80.11.3279, PMID: 6304704 PMC394024

[B72] Negi V. Miller A. S. Kuhn R. J. (2025). Advances in viroporin function and structure: A comparative analysis of alphavirus 6K with well-characterized viroporins. Viruses. 17, 868. doi: 10.3390/v17060868, PMID: 40573459 PMC12197650

[B48] Owen K. E. Kuhn R. J. (1997). Alphavirus budding is dependent on the interaction between the nucleocapsid and hydrophobic amino acids on the cytoplasmic domain of the E2 envelope glycoprotein. Virology. 230, 187–196. doi: 10.1006/viro.1997.8480, PMID: 9143274

[B71] Ramsey J. Mukhopadhyay S. (2017). Disentangling the frames, the state of research on the alphavirus 6K and TF Proteins. Viruses. 9, 228. doi: 10.3390/v9080228, PMID: 28820485 PMC5580485

[B69] Ramsey J. Renzi E. C. Arnold R. J. Trinidad J. C. Mukhopadhyay S. (2017). Palmitoylation of Sindbis virus TF protein regulates its plasma membrane localization and subsequent incorporation into virions. J. Virol. 91, e02000-16. doi: 10.1128/JVI.02000-16, PMID: 27852864 PMC5244351

[B41] Rayaprolu V. Moore A. Wang J. C. Goh B. C. Perilla J. Zlotnick A. . (2017). Length of encapsidated cargo impacts stability and structure of *in vitro* assembled alphavirus core-like particles. J. Phys. Condens Matter. 29, 484003. doi: 10.1088/1361-648X/aa90d0, PMID: 28975896 PMC7103146

[B17] Read C. M. Plante K. Rafael G. Rossi S. L. Braun W. Weaver S. C. . (2021). Designing multivalent immunogens for alphavirus vaccine optimization. Virology. 561, 117–124. doi: 10.1016/j.virol.2020.11.010, PMID: 33823988 PMC8277671

[B59] Ruiz-Guillen M. Gabev E. Smerdou C. (2016). Capsid-deficient alphaviruses generate propagative infectious microvesicles at the plasma membrane. Cell. Mol. Life Sci. 73, 3897–3916. doi: 10.1007/s00018-016-2230-1, PMID: 27117550 PMC7079800

[B39] Rümenapf T. Brown D. T. Strauss E. G. König M. Rameriz-Mitchel R. Strauss J. H. (1995). Aura alphavirus subgenomic RNA is packaged into virions of two sizes. J. Virol. 69, 1741–1746. doi: 10.1128/jvi.69.3.1741-1746.1995, PMID: 7853512 PMC188778

[B14] Schuffenecker I. Iteman I. Michault A. Murri S. Frangeul L. Vaney M. C. . (2006). Genome microevolution of chikungunya viruses causing the Indian Ocean outbreak. PloS Med. 3, e263. doi: 10.1371/journal.pmed.0030263, PMID: 16700631 PMC1463904

[B97] Sengupta R. Mihelc E. M. Angel S. Lanman J. K. Kuhn R. J. Stahelin R. V. (2022). Contribution of the Golgi apparatus in the morphogenesis of a virus-induced cytopathic vacuolar system. Life Sci. Alliance. 5, e202000887. doi: 10.26508/lsa.202000887, PMID: 36137747 PMC9500387

[B15] Silva L. A. Dermody T. S. (2017). Chikungunya virus: Epidemiology, replication, disease mechanisms, and prospective intervention strategies. J. Clin. Investig. 127, 737–749. doi: 10.1172/JCI84417, PMID: 28248203 PMC5330729

[B58] Skoging-Nyberg U. Liljestrom P. (2001). M-X-I motif of Semliki Forest virus capsid protein affects nucleocapsid assembly. J. Virol. 75, 4625–4632. doi: 10.1128/JVI.75.10.4625-4632.2001, PMID: 11312332 PMC114215

[B54] Skoging U. Vihinen M. Nilsson L. Liljestrom P. (1996). Aromatic interactions define the binding of the alphavirus spike to its nucleocapsid. Structure. 4, 519–529. doi: 10.1016/S0969-2126(96)00058-5, PMID: 8736551

[B50] Snyder J. E. Berrios C. J. Edwards T. J. Jose J. Perera R. Kuhn R. J. (2012). Probing the early temporal and spatial interaction of the Sindbis virus capsid and E2 proteins with reverse genetics. J. Virol. 86, 12372–12383. doi: 10.1128/JVI.01220-12, PMID: 22951842 PMC3486501

[B62] Snyder A. J. Mukhopadhyay S. (2012). The alphavirus E3 glycoprotein functions in a clade-specific manner. J. Virol. 86, 13609–13620. doi: 10.1128/JVI.01805-12, PMID: 23035234 PMC3503070

[B37] Sokoloski K. J. Nease L. M. May N. A. Gebhart N. N. Jones C. E. Morrison T. E. . (2017). Identification of interactions between sindbis virus capsid protein and cytoplasmic vRNA as novel virulence determinants. PloS Pathog. 13, e1006473. doi: 10.1371/journal.ppat.1006473, PMID: 28662211 PMC5507600

[B73] Sokoloski K. J. Snyder A. J. Liu N. H. Hayes C. A. Mukhopadhyay S. Hardy R. W. (2013). Encapsidation of host-derived factors correlates with enhanced infectivity of Sindbis virus. J. Virol. 87, 12216–12226. doi: 10.1128/JVI.02437-13, PMID: 24006438 PMC3807880

[B23] Song H. Zhao Z. Chai Y. Jin X. Li C. Yuan F. . (2019). Molecular basis of arthritogenic alphavirus receptor MXRA8 binding to chikungunya virus envelope protein. Cell 177, 1714–1724.e12. doi: 10.1016/j.cell.2019.04.008, PMID: 31080063

[B52] Soonsawad P. Xing L. Milla E. Espinoza J. M. Kawano M. Marko M. . (2010). Structural evidence of glycoprotein assembly in cellular membrane compartments prior to alphavirus budding. J. Virol. 84, 11145–11151. doi: 10.1128/JVI.00036-10, PMID: 20739526 PMC2953181

[B47] Suomalainen M. Liljestrom P. Garoff H. (1992). Spike protein-nucleocapsid interactions drive the budding of alphaviruses. J. Virol. 66, 4737–4747. doi: 10.1128/jvi.66.8.4737-4747.1992, PMID: 1629953 PMC241300

[B61] Taylor G. M. Hanson P. I. Kielian M. (2007). Ubiquitin depletion and dominant-negative VPS4 inhibit rhabdovirus budding without affecting alphavirus budding. J. Virol. 81, 13631–13639. doi: 10.1128/JVI.01688-07, PMID: 17913808 PMC2168838

[B4] Tesh R. B. Watts D. M. Russell K. L. Damodaran C. Calampa C. Cabezas C. . (1999). Mayaro virus disease: an emerging mosquito-borne zoonosis in tropical south america. Clin. Infect. Dis. 28, 67–73. doi: 10.1086/515070, PMID: 10028074

[B60] Torii S. Orba Y. Sasaki M. Tabata K. Wada Y. Carr M. . (2020). Host ESCRT factors are recruited during chikungunya virus infection and are required for the intracellular viral replication cycle. J. Biol. Chem. 295, 7941–7957. doi: 10.1074/jbc.RA119.012303, PMID: 32341071 PMC7278350

[B63] Uchime O. Fields W. Kielian M. (2013). The role of E3 in pH protection during alphavirus assembly and exit. J. Virol. 87, 10255–10262. doi: 10.1128/JVI.01507-13, PMID: 23864626 PMC3754015

[B78] Ventoso I. Berlanga J. J. Toribio R. Díaz-López I. (2024). Translational control of alphavirus–host interactions: implications in viral evolution, tropism and antiviral response. Viruses. 16, 205. doi: 10.3390/v16020205, PMID: 38399981 PMC10893052

[B33] Volkova E. Gorchakov R. Frolov I. (2006). The efficient packaging of Venezuelan equine encephalitis virus-specific RNAs into viral particles is determined by nsP1–3 synthesis. Virology. 344, 315–327. doi: 10.1016/j.virol.2005.09.010, PMID: 16239019 PMC2430184

[B21] Voss J. Vaney M. C. Duquerroy S. Vonrhein C. Girard-Blanc C. Crublet E. . (2010). Glycoprotein organization of Chikungunya virus particles revealed by X-ray crystallography. Nature. 468, 709–712. doi: 10.1038/nature09555, PMID: 21124458

[B80] Wang N. Merits A. Veit M. Lello L. S. Kong S. Jiao H. . (2024). LDL receptor in alphavirus entry: structural analysis and implications for antiviral therapy. Nat. Commun. 15, 4906. doi: 10.1038/s41467-024-49301-1, PMID: 38851803 PMC11162471

[B32] Warrier R. Linger B. R. Golden B. L. Kuhn R. J. (2008). Role of sindbis virus capsid protein region II in nucleocapsid core assembly and encapsidation of genomic RNA. J. virology. 82, 4461–4470. doi: 10.1128/JVI.01936-07, PMID: 18305029 PMC2293040

[B1] Weaver S. C. Frolov I. (2005). “ Togaviruses,” in Virology, vol. 2 . Eds. Mahy B. W. J. Meulen V. T. ( ASM Press, Salisbury, United Kingdom), 1010–1024. doi:

[B12] Weaver S. C. Winegar R. Manger I. D. Forrester N. L. (2012). Alphaviruses: Population genetics and determinants of emergence. Antiviral Res. 94, 242–257. doi: 10.1016/j.antiviral.2012.04.002, PMID: 22522323 PMC3737490

[B66] Wu S.-R. Haag L. Hammar L. Wu B. Garoff H. Xing L. . (2007). The dynamic envelope of a fusion class II virus: PREFUSION STAGES OF SEMLIKI FOREST VIRUS REVEALED BY ELECTRON CRYOMICROSCOPY. J. Biol. Chem. 282, 6752–6762. doi: 10.1074/jbc.M609125200, PMID: 17192272

[B10] Zaid A. Burt F. J. Liu X. Poo Y. S. Zandi K. Suhrbier A. . (2021). Arthritogenic alphaviruses: Epidemiological and clinical perspective on emerging arboviruses. Lancet Infect. Dis. 21, e123–e133. doi: 10.1016/S1473-3099(20)30491-6, PMID: 33160445

[B68] Zhang X. Fugère M. Day R. Kielian M. (2003). Furin processing and proteolytic activation of semliki forest virus. J. Virol. 77, 2981–2989. doi: 10.1128/JVI.77.5.2981-2989.2003, PMID: 12584323 PMC149766

[B65] Zhang R. Hryc C. F. Cong Y. Liu X. Jakana J. Gorchakov R. . (2011). 4.4 Å Cryo-EM structure of an enveloped alphavirus Venezuelan equine encephalitis virus. EMBO J. 30, 3854–3863. doi: 10.1038/emboj.2011.261, PMID: 21829169 PMC3173789

[B100] Zhang Z. Huang J. Li Z. Deng C. Zhang H. Zhang B. . (2025). An alphavirus vaccine development utilizing RNA replication-defective strategy. Mol. Ther. 33, 6282–6297. doi: 10.1016/j.ymthe.2025.08.051, PMID: 40898615 PMC12703151

[B67] Zhang X. Kielian M. (2004). Mutations that promote furin-independent growth of semliki forest virus affect P62-E1 interactions and membrane fusion. Virology. 327, 287–296. doi: 10.1016/j.virol.2004.06.037, PMID: 15351216

[B84] Zhao H. Lindqvist B. Garoff H. von Bonsdorff C. H. Liljeström P. (1994). A tyrosine-based motif in the cytoplasmic domain of the alphavirus envelope protein is essential for budding. EMBO J. 13, 4204–4211. doi: 10.1002/j.1460-2075.1994.tb06740.x, PMID: 7925266 PMC395347

